# Meta-Analysis Reveals the Association of Common Variants in the *Uncoupling Protein* (UCP) *1–3* Genes with Body Mass Index Variability

**DOI:** 10.1371/journal.pone.0096411

**Published:** 2014-05-07

**Authors:** Letícia A. Brondani, Tais S. Assmann, Bianca M. de Souza, Ana P. Bouças, Luis H. Canani, Daisy Crispim

**Affiliations:** 1 Endocrinology Division, Hospital de Clínicas de Porto Alegre, Universidade Federal do Rio Grande do Sul, Porto Alegre, Rio Grande do Sul, Brazil; 2 Postgraduate Program in Medical Sciences, Endocrinology, Universidade Federal do Rio Grande do Sul, Porto Alegre, Rio Grande do Sul, Brazil; Monash University, Australia

## Abstract

**Background:**

The relationship between uncoupling protein (*UCP*) *1–3* polymorphisms and susceptibility to obesity has been investigated in several genetic studies. However, the impact of these polymorphisms on obesity is still under debate, with contradictory results being reported. Until this date, no meta-analysis evaluated the association of *UCP* polymorphisms with body mass index (BMI) variability. Thus, this paper describe a meta-analysis conducted to evaluate if the -3826A/G (*UCP1*); -866G/A, Ala55Val and Ins/Del (*UCP2*) and -55C/T (*UCP3*) polymorphisms are associated with BMI changes.

**Methods:**

A literature search was run to identify all studies that investigated associations between *UCP1-3* polymorphisms and BMI. Weighted mean differences (WMD) were calculated for different inheritance models.

**Results:**

Fifty-six studies were eligible for inclusion in the meta-analysis. Meta-analysis results showed that *UCP2* 55Val/Val genotype was associated with increased BMI in Europeans [Random Effect Model (REM) WMD 0.81, 95% CI 0.20, 1.41]. Moreover, the *UCP2* Ins allele and *UCP3-*55T/T genotype were associated with increased BMI in Asians [REM WMD 0.46, 95% CI 0.09, 0.83 and Fixed Effect Model (FEM) WMD 1.63, 95% CI 0.25, 3.01]. However, a decreased BMI mean was observed for the *UCP2-*866 A allele in Europeans under a dominant model of inheritance (REM WMD −0.18, 95% CI −0.35, −0.01). There was no significant association of the *UCP1-*3826A/G polymorphism with BMI mean differences.

**Conclusions:**

The meta-analysis detected a significant association between the *UCP2-*866G/A, Ins/Del, Ala55Val and *UCP3-*55C/T polymorphisms and BMI mean differences.

## Introduction

Obesity is the most common nutritional disease in industrialized countries, constituting a priority health problem. It is associated with the development of cardiovascular diseases, type 2 diabetes mellitus and various cancers, which leads to higher morbidity and mortality rates, reducing the quality and expectancy of life of sufferers [1]. Obesity is characterized by an excessive accumulation of body fat resulting from an imbalance between energy intake and expenditure [2]. This imbalance can be due to genetic or environmental risk factors, or more likely due to a combination of both, an intertwining of genetics and environment [3]. A general consensus is that about 65% of variation in obesity is genetic [4], and candidate genes include those that control energy expenditure and thermogenesis, such as those encoding mitochondrial uncoupling proteins (UCPs) [5].

Uncoupling proteins 1, 2 and 3 are members of an anion-carrier protein family located in the mitochondrial inner membrane [5]–[7]. These proteins share structural similarities, but have different tissue expressions [7]. The original UCP, UCP1, is mainly expressed in brown adipose tissue [8], [9]. Uncoupling protein 2 is distributed across a wide range of tissue and cell types, whereas UCP3 is mainly restricted to skeletal muscle [9]. Several studies have shown that UCPs decrease metabolic efficiency by uncoupling substrate oxidation in mitochondria from ATP synthesis by the mitochondrial respiratory chain. This is probably accomplished by promoting net translocation of protons from the intermembrane space, across the inner mitochondrial membrane, to the mitochondrial matrix, thereby dissipating the potential energy available for ATP synthesis, and consequently, decreasing ATP production. This uncoupling effect leads to homologue- and tissue-specific functions, such as thermogenesis and energy expenditure (UCP1), regulation of free-fatty acids (FFAs) metabolism (UCP2 and UCP3), reduction in ROS formation (UCP1-3), and regulation of ATP-dependent processes (UCP2) [5], [6], [9], [10].

The relationship between *UCP1-3* loci and susceptibility to obesity has been investigated in several genetic studies and particular attention has been focused on the -3826A/G (rs1800592) polymorphism in the *UCP1* gene, the -866G/A polymorphism (rs659366), the Ala55Val (C/T; rs660339) polymorphism and the Ins/Del polymorphism in the *UCP2* gene, and the -55C/T (rs1800849) polymorphism in the *UCP3* gene [5], [10], [11]. However, the impact of these polymorphisms on obesity susceptibility is still under debate, with contradictory results being reported. In this context, Qian *et al*. [12] evaluated the association of the *UCP2-*886G/A, *UCP2* Ala55Val, and *UCP3-*55C/T polymorphisms with obesity in a meta-analysis of 22 articles. The meta-analysis revealed that the *UCP2-*866G/A polymorphism may be a risk factor for obesity in Europeans, but not in Asian subjects. In agreement with this data, Andersen *et al*. [13] published a meta-analysis showing an association between the *UCP2-*866G/A polymorphism and obesity in subjects of European descent. Until this date, no meta-analysis evaluated the association of *UCP* polymorphisms with body mass index (BMI) variability. Thus, to further investigate the potential role of *UCP1-3* polymorphisms in influencing BMI changes, we performed a systematic review and meta-analysis of the literature on the subject.

## Materials and Methods

### Search Strategy, Eligibility Criteria and Data Extraction

This study was designed and described in accordance with current guidelines for execution of systematic reviews and meta-analyses [14], [15]. Both PubMed and Embase repositories were searched systemically to identify all available genetic studies which analyzed associations between obesity and the most-often studied polymorphisms of *UCP1-3* genes: -3826A/G (rs1800592) polymorphism in the promoter region of the *UCP1* gene, -866G/A (rs659366) polymorphism in the promoter region, the Ala55Val (C/T; rs660339) polymorphism in exon 4 and the Ins/Del polymorphism, which is an insertion/deletion of 45 bp in the 3′ untranslated region (3′UTR) of exon 8 of the *UCP2* gene, and the -55C/T (rs1800849) polymorphism in the promoter region of the *UCP3* gene. The following medical subject headings (MeSH) were searched: (‘‘Obesity’’ OR “Body mass index”) AND (‘‘mitochondrial uncoupling protein’’ OR ‘‘SLC25A27 protein, human’’ OR ‘‘mitochondrial uncoupling protein 2″ OR ‘‘mitochondrial uncoupling protein 3″) AND (‘‘mutation’’ OR ‘‘frameshift mutation’’ OR ‘‘germ-line mutation’’ OR ‘‘INDEL mutation’’ OR ‘‘mutation, missense’’ OR ‘‘point mutation’’ OR ‘‘codon, nonsense’’ OR ‘‘sequence deletion’’ OR ‘‘polymorphism, genetic’’ OR ‘‘polymorphism, single nucleotide’’ OR ‘‘polymorphism, restriction fragment length’’). The search was limited to human studies and English or Spanish language papers and was completed on January, 2014. All of the articles identified were also searched manually to identify any other relevant citations.

Two investigators (B.M.S and A.P.B.) independently reviewed the titles and abstracts of all articles selected in order to evaluate whether the studies were eligible for inclusion in the meta-analysis. Disagreements were resolved by discussion between them and when necessary a third reviewer (D.C.) was consulted. When abstracts did not provide sufficient information to fulfill the inclusion and exclusion criteria, the full text of the article was retrieved for evaluation. We included observational studies that compared BMI (mean ± SD) among different genotypes of one or more of the *UCP1-3* polymorphisms in question. Studies were excluded from the analysis if they did not have sufficient data or if they did not employ validated genotyping methods. If data were duplicated and had been published more than once, the most complete study was chosen.

Data were independently extracted by two investigators (L.A.B. and T.S.A.) using a standardized abstraction form, and consensus was sought in all extracted items. When consensus could not be reached, differences in data extraction were resolved by referencing the original publication or by consulting a third reviewer (D.C.). The information extracted from each individual study was as follows: name of first author, publication year, ethnicity, number of subjects, age, gender, and BMI in different genotypes of *UCP1-3* polymorphisms.

### Statistical Analyses for Meta-analyses

Subjects’ genotype distributions were tested for conformity with Hardy-Weinberg Equilibrium (HWE) using a goodness-of-fitness χ^2^ test. Some articles only reported the mean BMI according to the different genotypes after stratification by sex or other characteristic such as presence of diabetes or obesity, so we had to calculate the pooled mean BMI to include this information in our meta-analysis. Heterogeneity among studies was tested using a χ^2^-based Cochran’s *Q* statistic and inconsistency was assessed with the I^2^ metric [16], [17]. Heterogeneity was considered statistically significant at P<0.10 for the *Q* statistic and I^2^>50% for the I^2^ metric statistics. Where significant heterogeneity was detected, the DerSimonian and Laird random effect model (REM) was used to calculate weighted mean differences (95% CI) in BMI for each individual study and for the pooled effect; where heterogeneity was not significant, the fixed effect model (FEM) was used for this calculation [16], [17]. Weighted mean differences (95% CI) in BMI were evaluated for additive, recessive and dominant models of inheritance [18], [19].

Meta-regression and sensitivity analyses were carried out to identify key studies with a substantial impact on inter-study heterogeneity. The factors investigated by meta-regression were age, gender and ethnicity. Sensitivity analyses were performed after stratifying the studies by ethnicity, given that the *UCP* polymorphisms show variable frequencies across ethnic groups. Risk of publication bias was assessed using funnel plot graphics, analyzed both visually and with the Begg and Egger test [20]. The significance of the intercept was determined by the t test, as proposed by Egger, with P<0.10 considered indicative of statistically significant publication bias. All statistical analyses were performed using Stata 11.0 software (StataCorp, College Station, TX, USA).

## Results

### Literature Search and Characteristics of Eligible Studies

**Figure 1:**
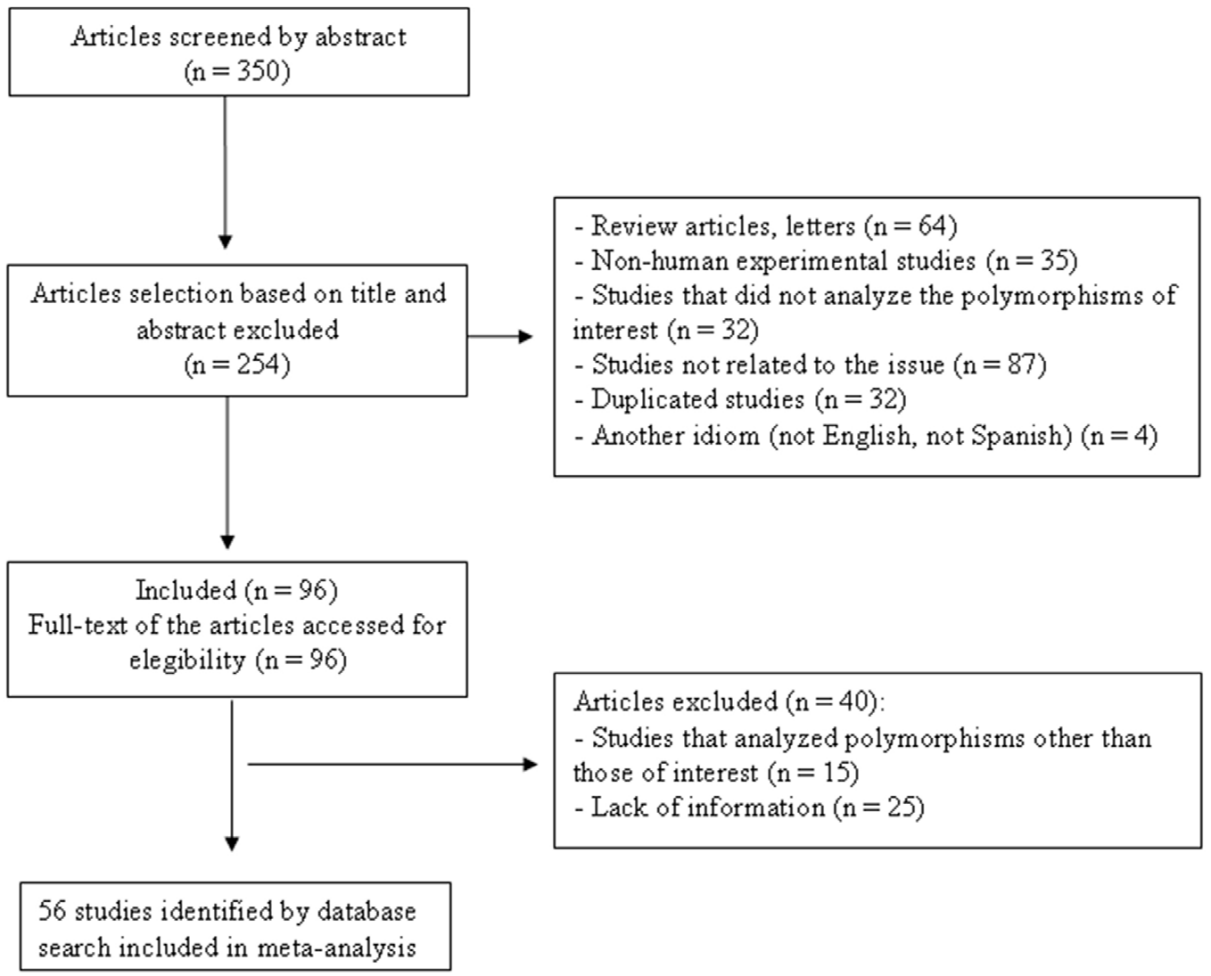
Flowchart illustrating the search strategy used to identify association studies of *UCP1-3* polymorphisms with body mass index changes for inclusion in the meta-analysis.

**Figure 1** is a flow diagram illustrating the strategy used to identify and select studies for inclusion in the meta-analysis. A total of 350 potentially relevant citations were retrieved by searching the electronic databases and 254 of them were excluded during the review of titles and abstracts. Ninety-six articles therefore appeared to be eligible at this point and their full texts were evaluated. However, after careful analysis of the full texts, another 40 studies were excluded because of missing information, ineligible study designs or because they genotyped other *UCP* polymorphisms, but not the ones of interest here. A total of 56 articles fulfilled the eligibility criteria and were included in the meta-analyses [21]–[77]. The available characteristics of studies included in primary analyses are shown in **Table S1**. The median age of analyzed subjects was 46.0 (minimum 8.4–maximum 65.5 values) years, and the median BMI was 37.0 (20.3–50.2). Sixty-one percent of the subjects were men, 25.3% had diabetes, and 57.1% had obesity. Most of the studies were performed in European populations.

**Table S2** shows the mean BMI ± SD according to the different *UCP1-3* genotypes in all studies included in the meta-analysis. Twenty studies analyzed the *UCP1-*3826A/G polymorphism (6191 subjects), 18 studies analyzed the *UCP2-*886G/A polymorphism (12072 subjects), 6 analyzed the *UCP2* Ala55Val polymorphism (15060 subjects), 8 analyzed the *UCP2* Ins/Del polymorphism (8490 subjects), and 8 investigated the *UCP3-*55C/T polymorphism (6663 subjects).

### Quantitative Synthesis

**Figure 2:**
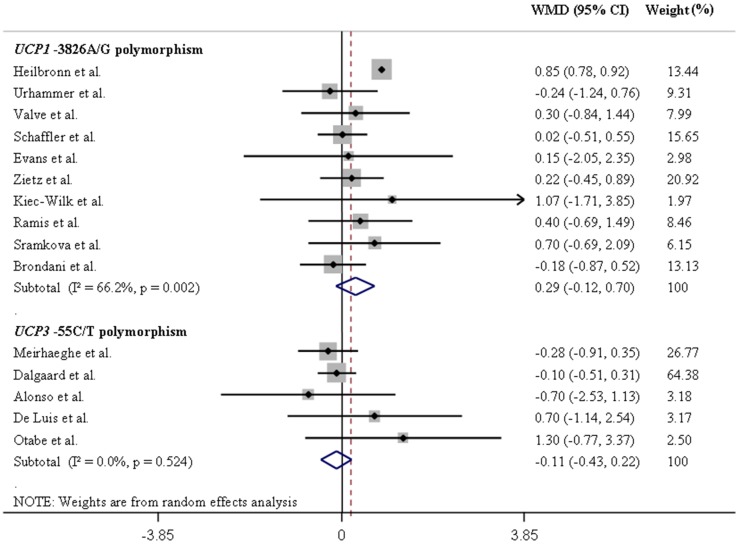
Forest plots showing individual and pooled weighted mean differences (95% CI) for the associations between *UCP1-*3826A/G and *UCP3-*55C/T polymorphisms and body mass index in Europeans, under a dominant inheritance model (A/A *vs*. G/G + A/G for the *UCP1-*3826A/G polymorphism, and C/C *vs*. T/T + C/T for the *UCP3-*55C/T polymorphism). The area of the squares reflects the study-specific weight. The diamond shows the summary random-effect weighted mean differences estimated from the studies.

**Figure 3:**
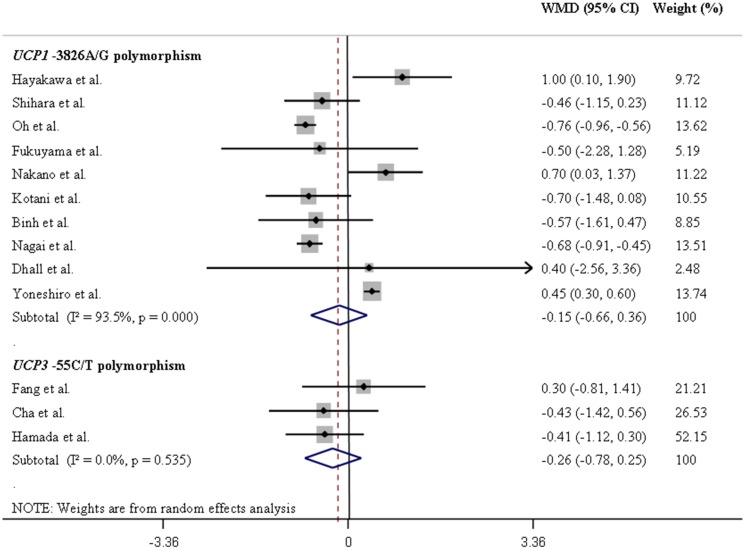
Forest plots showing individual and pooled weighted mean differences (95% CI) for the associations between *UCP1-*3826A/G and *UCP3-*55C/T polymorphisms and body mass index in Asians, under a dominant inheritance model (A/A *vs*. G/G + A/G for the *UCP1-*3826A/G polymorphism, and C/C *vs*. T/T + C/T for the *UCP3-*55C/T polymorphism). The area of the squares reflects the study-specific weight. The diamond shows the summary random-effect weighted mean differences estimated from the studies.

**Figure 4:**
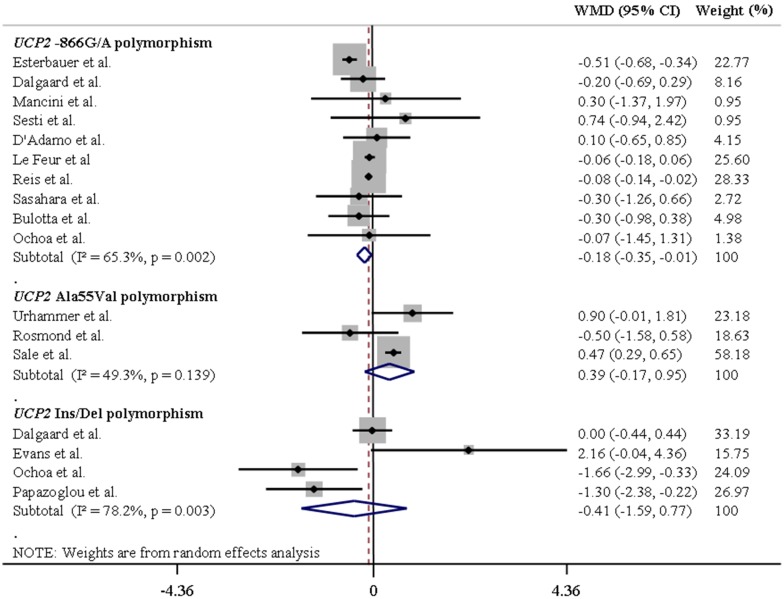
Forest plots showing individual and pooled weighted mean differences (95% CI) for the associations between *UCP2-*866G/A, Ala55Val and Ins/Del polymorphisms and body mass index in Europeans, under a dominant inheritance model (G/G *v*s. A/A + G/A for the *UCP2-*866G/A polymorphism, Ala/Ala *vs*. Val/Val + Ala/Val for the *UCP2* Ala55Val polymorphism, and Del/Del *vs*. Ins/Ins+Ins/Del for the *UCP2* Ins/Del polymorphism). The area of the squares reflects the study-specific weight. The diamond shows the summary random-effect weighted mean differences estimated from the studies.

**Figure 5:**
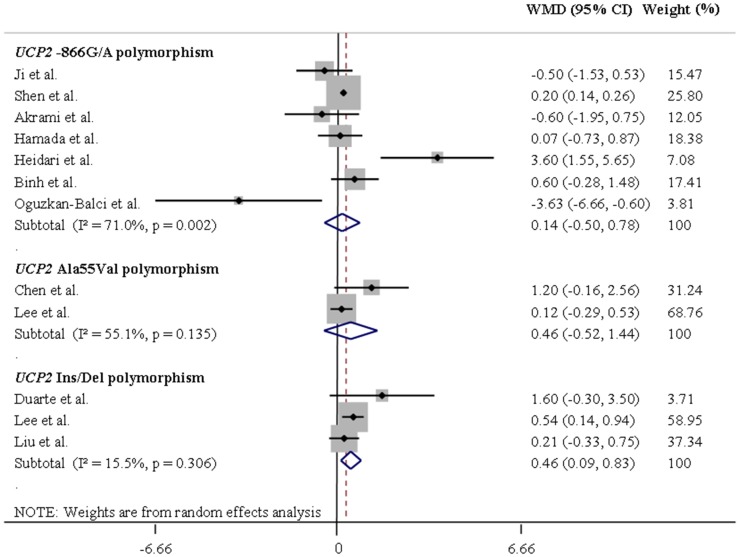
Forest plots showing individual and pooled weighted mean differences (95% CI) for the associations between *UCP2-*866G/A, Ala55Val and Ins/Del polymorphisms and body mass index in Asians, under a dominant inheritance model (G/G *v*s. A/A + G/A for the *UCP2-*866G/A polymorphism, Ala/Ala *vs*. Val/Val + Ala/Val for the *UCP2* Ala55Val polymorphism, and Del/Del *vs*. Ins/Ins + Ins/Del for the *UCP2* Ins/Del polymorphism). The area of the squares reflects the study-specific weight. The diamond shows the summary random-effect weighted mean differences estimated from the studies.

**Table 1** summarizes the results of the pooled analyses for the associations between *UCP* polymorphisms and differences in mean BMI values. **Figure 2** and **Figure 3** illustrate pooled WMD in BMI values for the *UCP1-*3826A/G and *UCP3-*55C/T polymorphisms in Europeans and Asians, respectively, assuming a dominant inheritance model. **Figure 4** and **Figure 5** show pooled WMD in BMI for the *UCP2-*866G/A, Ala55Val and Ins/Del polymorphisms in Europeans and Asians, respectively, also under a dominant model.

**Table 1:** Pooled measures for associations between *UCP1-*3826A/G, *UCP2-*866G/A, *UCP2* Ala55Val, *UCP2* Ins/Del and *UCP3-*55C/T polymorphisms and body mass index mean differences.

Inheritance model	n studies	n patients	I^2^ %	WMD (95% CI)	Sensitivity analysis	
					I^2^ %	WMD (95% CI)
***UCP1-*** **3826 A/G**						
Additive	17	3578	93.8	0.07 (−0.48, 0.63)		
Asian	9	888	95.0	−0.11 (−0.78, 0.59)		
European	8	2690	76.7	0.34 (−0.61, 1.29)	0.0	−0.02 (−0.58, 0.53)^a^
Recessive	17	5909	91.0	0.12 (−0.30, 0.54)		
Asian	9	1620	94.1	0.00 (−0.53, 0.54)		
European	8	4289	68.4	0.29 (−0.51, 1.09)	0.0	−0.02 (−0.56, 0.53)^a^
Dominant	20	6191	93.3	0.05 (−0.33, 0.42)		
Asian	10	1713	93.5	−0.15 (−0.66, 0.36)		
European	10	4478	59.1	0.29 (−0.12, 0.70)	0.0	0.09 (−0.20, 0.39)^a^
***UCP2-*** **866 G/A**						
Additive	16	7125	99.4	−0.07 (−0.52, 0.37)		
Asian	6	1440	72.9	0.37 (−0.47, 1.22)		
European	10	5685	98.6	−0.30 (−1.02, 0.41)		
Recessive	17	12455	99.4	−0.16 (−1.07, 0.76)		
Asian	6	2794	98.7	−1.07 (−4.39, 2.25)		
European	11	9661	98.7	0.44 (−0.27, 1.15)		
Dominant	17	10471	85.3	−0.07 (−0.26, 0.11)		
Asian	7	2894	71.0	0.14 (−0.50, 0.78)		
European	10	7577	65.3	−0.18 (−0.35, −0.01)	0.0	−0.08 (−0.13, −0.02)^b^
***UCP2*** ** I/D**						
Additive	5	5796	68.4	0.22 (−1.20, 1.64)		
European	3	5072	82.3	−0.07 (−2.79, 2.66)		
Recessive	5	5796	53.8	0.19 (−0.94, 1.31)	0.0	0.67 (−0.06, 1.29)^c^
European	3	5072	76.2	−0.05 (−2.31, 2.21)	0.0	0.75 (−0.02, 1.52)^c^
Dominant	8	8490	78.1	0.24 (−0,41, 0.89)		
Asian	3	3508	15.5	0.46 (0.09, 0.83)		
European	4	4893	78.2	−0.41 (−1.59, 0.77)		
***UCP2*** ** Ala55val**						
Additive	5	7488	92.7	0.39 (−0.32, 1.10)		
European	3	894	70.2	0.74 (−0.24, 1.72)		
Recessive	6	17002	96.6	0.48 (0.01, 0.95)		
European	4	3974	97.0	0.81 (0.20, 1.41)		
Dominant	6	15060	72.4	0.27 (−0.06, 0.59)		
Asian	2	1276	55.1	0.46 (−0.52, 1.44)		
European	3	1728	49.3	0.39 (−0.17, 0.95)		
***UCP3-*** **55C/T**						
Additive	6	3568	71.9	0.46 (−0.77, 1.68)		
Asian	3	434	81.7	0.32 (−2.07, 2.71)	0.0	1.46 (0.02, 2.90)^c^
European	3	3134	64.6	0.62 (−0.92, 2.16)	0.0	0.26 (−0.43, 0.94)^d^
Recessive	6	6131	73.1	0.54 (−0.67, 1.76)		
Asian	3	796	83.8	0.46 (−1.98, 2.90)	0.0	1.63 (0.25, 3.01)^c^
European	3	5335	61.8	0.62 (−0.83, 2.07)	0.0	0.29 (−0.38, 0.96)^d^
Dominant	8	6663	0.0	−0.15 (−0.43, 0.12)		
Asian	3	796	0.0	−0.26 (−0.78, 0.25)		
European	5	5867	0.0	−0.11 (−0.43, 0.22)		

Sensitivity analysis was conducted to reduce heterogeneity by omitting studies if I^2^≥50%. Articles excluded after sensitivity analysis: Heilbron *et al*. [26] ^a^;

Esterbauer *et al*. [39] ^b^,

Hamada *et al*. [70] ^c^ and Otabe *et al*. [65] ^d^.

WMD = weighted mean difference.

*UCP1-*3826A/G and *UCP3-*55C/T polymorphisms were not associated with any change in BMI irrespectively of the inheritance model or after stratification by ethnicity (**Table 1**). The A allele of the *UCP2-*866G/A polymorphism was significantly associated with decreased WMD in BMI in Europeans (REM WMD = −0.18, 95% CI −0.35 – −0.01, for the dominant model) but not in Asians. On the other hand, the Ins allele of the *UCP2* Ins/Del polymorphism was associated with increased WMD in BMI in Asians (REM WMD = 0.46, 95% CI 0.09−0.83, dominant model) but not in Europeans. The *UCP2* Ala55Val polymorphism was also associated with increased WMD in BMI in Europeans, under a recessive model (REM WMD = 0.81, 95% CI 0.20−1.41; Ala/Ala **+** Ala/Val *vs*. Val/Val).

As can be observed in **Table 1**, there were significant heterogeneities (I^2^>50%) among studies in almost all comparisons of BMI according to the *UCP1-3* genotypes under the different inheritance models. To investigate this finding in greater depth, sex, age and ethnicity were used as covariates in meta-regression analyses performed for the five *UCP* polymorphisms under different inheritance models. None of the covariates used in univariate and multivariate meta-regression analyses could individually explain the heterogeneities observed (data not shown).

Sensitivity analyses were carried out in order to estimate the influence of each individual study in the meta-analysis results. This was performed by repeating the meta-analyses omitting a different study each time. No individual study significantly influenced the inter-study heterogeneity for the *UCP2* Ala55Val polymorphism (data not shown). In contrast, some studies seem to be responsible for the inter-study heterogeneity observed in the meta-analyses of *UCP1-*3826A/G, *UCP2-*866G/A and Ins/Del, and *UCP3-*55C/T polymorphisms (**Table 1**). Exclusion of these studies from the respective meta-analysis abolished the heterogeneity; however, the pooled WMD in BMI only was significantly altered after the exclusion of one specific study [70] from the analyses of the *UCP3-*55C/T polymorphism in Asians (WMD = 1.46, 95% CI 0.02−2.90, for the additive model; and WMD = 1.63, 95% CI 0.25−3.01, for the recessive model; **Table 1**).

Importantly, funnel plots and Egger’s tests were performed to detect publication bias of the literature, as illustrated in **Figures S1, S2 and S3**. Symmetrical funnel plots were generated for each of the five *UCP* polymorphisms tested in the additive, dominant and recessive inheritance models. Egger’s tests further confirmed no publication bias for any of the *UCP* polymorphisms analyzed, which suggests that our data are statistically reliable.

## Discussion

Uncoupling protein 1, UCP2 and UCP3 are candidate genes for obesity because they decrease mitochondrial membrane potential and mediate proton leak [5], [8], [9]. Mutations reducing the activity of these proteins could reduce energy expenditure by increasing coupling of oxidative phosphorylation, thereby contributing to BMI changes. These are the reasons why the roles played by *UCP1-*3826A/G, *UCP2-*866G/A, *UCP2* Ala55Val, *UCP2* Ins/Del and *UCP3-*55C/T polymorphisms in obesity risk and BMI changes have been extensively studied, although the results of these associations are still inconclusive (reviewed in [5], [6], [78]). Therefore, to further investigate if these *UCP* polymorphisms are associated with changes in BMI, we conducted a meta-analysis of 56 published articles from different populations.

Our results suggest that the *UCP1-*3826A/G polymorphism is not associated with BMI changes. The A allele of the *UCP2-*866G/A polymorphism was associated with a 0.18 reduction in BMI in Europeans. On the other hand, the Ins allele of the *UCP2* Ins/Del polymorphism was associated with an increase of 0.46 units in BMI in Asians, while the Val/Val genotype of the *UCP2* Ala55Val polymorphism was associated with an increase of 0.81 units in BMI in Europeans under a recessive model of inheritance. Moreover, the T/T genotype of the *UCP3-*55C/T polymorphism was associated with increased of 1.6 units in BMI in Asians but only after sensitivity analysis. These results show that associations of *UCP2* and *UCP3* polymorphisms with BMI changes are probably under an important influence of ethnicity. This may be explained by known differences in lifestyle and body weight distributions between Asian and European populations as well as by differences in the genotype frequencies of the analyzed polymorphisms between ethnicities. In this context, Luan *et al.* [79] found that effects of genetic polymorphisms on obesity and related-disorders could be changed by nutritional characteristic of the population. Thus, it might be possible that different diet patterns between European and Asian populations could modulate the effect of *UCP* polymorphisms in BMI changes.

It is worth noting that two previous meta-analyses investigated one or more of the *UCP* polymorphisms included in our meta-analysis, although only regarding their associations with susceptibility to obesity. Qian [12] *et al*. included 22 studies in the meta-analysis, and their results support an association between the A allele of the *UCP2-*866G/A polymorphism and protection for obesity in Europeans, which is in agreement with our data. However, they were not able to find any association of the *UCP2* Ala55Val and *UCP3-*55C/T polymorphisms with obesity, which is in conflict with our data. One possible explanation for this discrepancy is that the outcome of Qian *et al*. [12] was presence of obesity while our outcome was BMI. It is reasonable to suppose that *UCP* polymorphisms may be associated with small changes in BMI; therefore, analyzing BMI changes would have a higher power to detect smaller associations with obesity. Furthermore, our meta-analysis included 56 studies, whereas the meta-analysis of Qian *et al*. [12] included only 22 studies. Andersen *et al*. [13] performed a meta-analysis of 8 studies that analyzed the association between the *UCP2-*886G/A polymorphism and obesity. In agreement with our data, they reported an association between the *UCP2-*866A allele and protection for obesity in Europeans. Moreover, they also observed an association between *UCP2-*866G/A polymorphism and insulin resistance among 5781 middle-aged Danes and 377 young health Danes, where subjects carrying the G allele consistently had lower insulin sensitivity [13].

The *UCP2-*866G/A polymorphism seems to be involved in putative binding sites for specific transcription factors and, in fact, several studies have shown that this promoter variant changes reporter gene activity [39], [77]. However, data in human tissues are conflicting, reporting either increased or decreased *UCP2* mRNA levels being associated with the -866A allele (reviewed in [6]). In differentiated adipocytes, the A allele has a 22% more effective transcriptional activity [39]; therefore, the association of the *UCP2-*866A allele with decreased BMI in Europeans appears to be biologically plausible since an increased *UCP2* gene expression in adipocytes would be associated with increased energy expenditure.

Our data indicate that the *UCP2* Val55Val genotype is associated with the highest change in BMI (WMD = 0.81) in Europeans. However, this polymorphism causes a conservative amino acid change at position 55 of exon 4 and, until now, there had been no evidence that this alteration generates a functional change in the protein [6]. It is therefore possible that this polymorphism may not be a true disease-causing variant, but could simply be reflecting the effects of a functional polymorphism elsewhere in the *UCP2/UCP3* loci. The biological significance of the *UCP2* Ins/Del polymorphism is not well known. However, its location in the 3′UTR suggests that this polymorphism could alter the mRNA processing or the stability of the transcript. Any reduction in the *UCP2* mRNA stability could compromise the ability to remove excess calories through thermogenesis, especially in a person with a propensity to obesity from other genetic or environmental influences [10]. This hypothesis is in agreement with our data showing that the *UCP2* Ins/Del polymorphism is associated with increased BMI in Asians.

The *UCP3-*55C/T polymorphism is located 55 bp upstream of the most commonly used transcription initiation site of skeletal muscle [80], and thus appears to be functional. Here, this polymorphism was associated with a significant increase in BMI in Asians but only after sensitivity analysis. Thus, this data should be interpreted with caution since was based in only two studies. Further studies are still needed to elucidate the functional effects of the *UCP3-*55C/T polymorphism on *UCP3* expression as well as to confirm its association with obesity. It is worth noting that a previous meta-analysis from our group showed an association of the *UCP3-*55C/T polymorphism with risk for type 2 diabetes mellitus in Asians (OR = 1.22, 95% CI 1.04–1.44, allele contrast model) [81], further indicating a role for this polymorphism in obesity-related features.

The results of the present meta-analysis should be interpreted within the context of a few limitations. First, meta-analysis is prone to publication bias, and although we have attempted to trace unpublished studies, we cannot be sure that small negative studies were overlooked. Moreover, we did not detect any significant publication bias in our analysis, showing that our data is statistically reliable. Second, because of the difficulty in getting the full texts of articles published in several languages, we only included studies published in English and Spanish. Third, inter-studies heterogeneity is common in meta-analyses for genetic association studies [82] and this can be a significant problem when interpreting their results. Our meta-analysis showed significant inter-study heterogeneity for most of the *UCP* polymorphisms analyzed. To investigate this problem in greater depth, meta-regression analyses were performed and showed that age, gender and ethnicity did not make significant contributions to inter-studies heterogeneity. The heterogeneity observed could be due to differences in sample selection, genotyping methods or gene-environment interactions and, without detailed information on the metabolic and clinical characteristics of the studies reviewed; we could not fully exclude the possibility that the heterogeneity observed might reduce our power to detect true associations. “Leave one out” sensitivity analyses showed that after excluding some studies, the heterogeneity for these analyses decreased significantly. However, despite the exclusion of these studies, only the pooled WMD obtained for the *UCP3-*55C/T polymorphism changed significantly: after the exclusion of Hamada *et al*. [70], the *UCP3-*55C/T polymorphism became associated with increase BMI in Asians. Fourth, obesity is a complex disorder that involves interactions of genes and environment. Many studies revealed different effects of the *UCP1-3* polymorphisms depending on the physical activity and lifestyles [83]–[85]; however, we had insufficient data to take these confounder factors into account in our meta-analysis. Fifth, we also cannot rule out the possibility of type II error when analyzing associations of the *UCP* polymorphisms and BMI changes after stratifying by ethnicity. For the whole sample, we had at least 80% power (α = 0.05) to detect even modest changes in WMD for all analyzed polymorphisms, which is an evidence that our results are robust.

In conclusion, our results indicate that the *UCP1-*3826A/G polymorphism is not associated with significant changes in BMI. However, our results suggest that the *UCP2-*866A allele is associated with decreased BMI in Europeans, while the *UCP2* Val55Val genotype is associated with increased BMI in Europeans. The *UCP2* Ins allele and the *UCP3-*55T/T genotype seem to be associated with increased BMI in Asians. It is important to keep in mind that BMI is one phenotype of obesity; thus, we can not exclude the possibility that these polymorphisms could have different effects in other phenotypes, such as fat body mass index, waist circumference, and intra-abdominal adipose tissue. Since small sample sizes were obtained for some of the analyses performed in Asians, further additional larger studies that allow stratification for ethnicity and gene-gene and gene-environment interactions should also be conducted in order to elucidate the roles possibly played by *UCP* polymorphisms in obesity and BMI changes.

## Supporting Information

Figure S1 **Funnel plot for studies of the** ***UCP1-*** **3826A/G polymorphism under a dominant model of inheritance.**
(TIF)

Figure S2 **Funnel plot for studies of the** ***UCP2-*** **866G/A, Ala55Val and Ins/Del polymorphisms under a dominant model of inheritance.**
(TIF)

Figure S3 **Funnel plot for studies of the** ***UCP3-*** **55C/T polymorphism under a dominant model of inheritance.**
(TIF)

Table S1 **Characteristics of the studies included in the meta-analyses.**
(XLS)

Table S2 **Body mass index means according to different genotypes of** ***UCP1-*** **3826A/G,** ***UCP2-*** **886G/A,** ***UCP2*** **Ala55Val,** ***UCP2*** **Ins/del and** ***UCP3-*** **55C/T polymorphisms for the studies included in the meta-analysis.**
(DOC)

Checklist S1 **PRISMA Checklist.**
(DOC)

Flow Diagram S1 **PRISMA Flow Diagram.**
(DOC)
