# Supplementary figures and images for: Meta-Analysis Reveals the Association of Common Variants in the Uncoupling Protein (UCP) 1–3 Genes with Body Mass Index Variability
Source: PLoS One. 2014 May 7;9(5):e96411. doi: 10.1371/journal.pone.0096411 (PMC4013025; doi:10.1371/journal.pone.0096411)

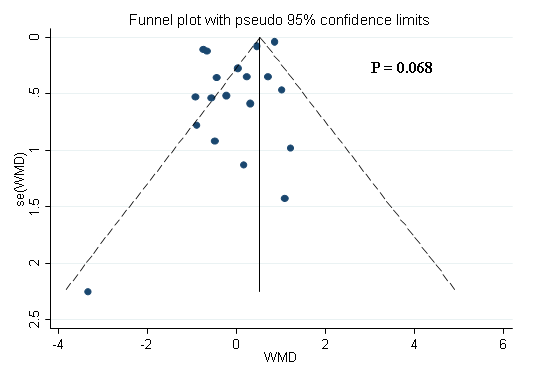

Supplement: Figure S1 — Funnel plot for studies of the UCP1- 3826A/G polymorphism under a dominant model of inheritance. (TIF) [file pone.0096411.s001.tif]

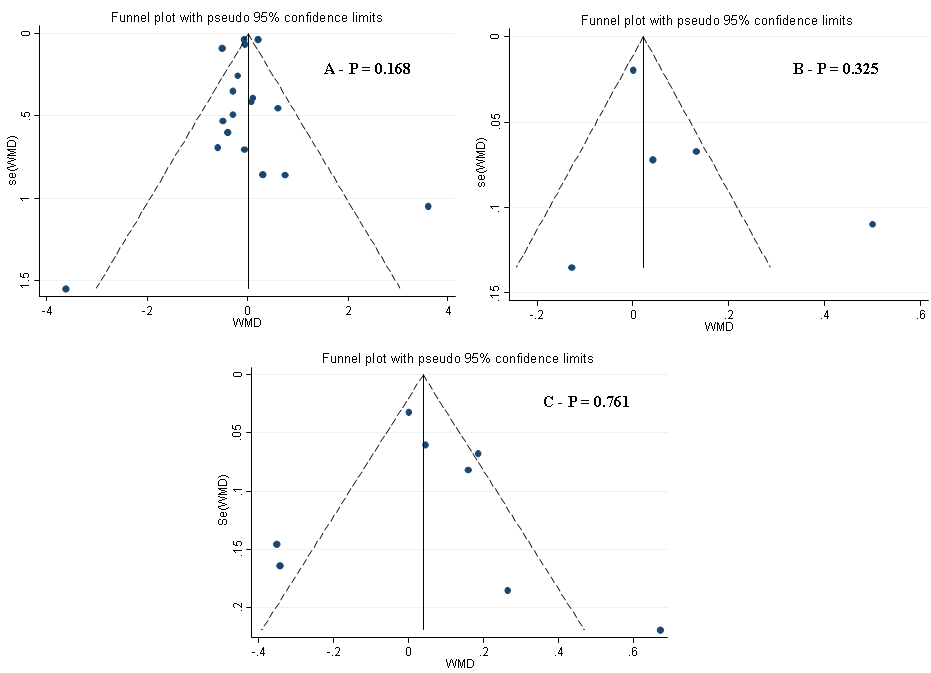

Supplement: Figure S2 — Funnel plot for studies of the UCP2- 866G/A, Ala55Val and Ins/Del polymorphisms under a dominant model of inheritance. (TIF) [file pone.0096411.s002.tif]

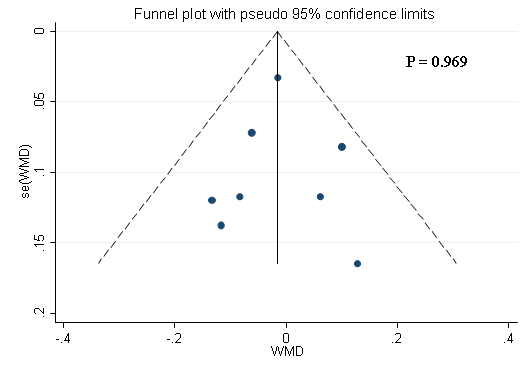

Supplement: Figure S3 — Funnel plot for studies of the UCP3- 55C/T polymorphism under a dominant model of inheritance. (TIF) [file pone.0096411.s003.tif]
