# Supplementary material for: Meta-Analysis Reveals the Association of Common Variants in the Uncoupling Protein (UCP) 1–3 Genes with Body Mass Index Variability
Source: PLoS One. 2014 May 7;9(5):e96411. doi: 10.1371/journal.pone.0096411 (PMC4013025; doi:10.1371/journal.pone.0096411)
Supplement: Table S2 — Body mass index means according to different genotypes of UCP1- 3826A/G, UCP2- 886G/A, UCP2 Ala55Val, UCP2 Ins/del and UCP3- 55C/T polymorphisms for the studies included in the meta-analysis. (DOC) [file pone.0096411.s005.doc]

**Table S1.** Body mass index means according to the genotypes of the *UCP1* -3826A/G, *UCP2* -866G/A, *UCP2* Ala55Val, *UCP2* Ins/Del and *UCP3* -55C/T polymorphisms for the studies included in the meta-analysis.

| ***UCP1* -3826A/G** |  |  |  | A/A |  |  | A/G |  |  | G/G |  |
| --- | --- | --- | --- | --- | --- | --- | --- | --- | --- | --- | --- |
| Reference | Year | Ethnicity | N | Mean | SD | N | Mean | SD | N | Mean | SD |
| Urhammer et al. | 1997 | European | 213 | 23.2 | 5.1 | 140 | 23.1 | 4.8 | 26 | 22.2 | 5.0 |
| Valve et al. | 1998 | European | 90 | 34.6 | 3.4 | 72 | 34.9 | 4.1 | 8 | 34.9 | 4.6 |
| Hayakawa et al. | 1999 | Asian | 56 | 23.0 | 3.1 | 106 | 24.2 | 2.6 | 52 | 23.6 | 2.4 |
| Schaffler et al. | 1999 | European | 581 | 25.5 | 4.2 | 361 | 25.5 | 4.4 | 78 | 25.6 | 3.6 |
| Evans et al. | 2000 | European | 314 | 36 | 13.9 | 245 | 36 | 13.5 | 44 | 37 | 14.9 |
| Heilbronn et al. | 2000 | European | 307 | 33.9 | 0.2 | 190 | 34.7 | 0.34 | 29 | 35.3 | 0.8 |
| Shihara et al. | 2001 | Asian | 89 | 21.9 | 2.9 | 191 | 21.5 | 2.8 | 71 | 21.3 | 2.6 |
| Zietz et al. | 2001 | European | 320 | 27.1 | 3.8 | 205 | 27.4 | 4.2 | 24 | 26.6 | 2.8 |
| Kiec-Wilk et al. | 2002 | European | 63 | 32.4 | 7.7 | 38 | 33.5 | 7.61 | 17 | 33.5 | 8.3 |
| Oh et al. | 2004 | Asian | 42 | 34.5 | 0.6 | 102 | 33.4 | 0.37 | 46 | 34.4 | 0.6 |
| Ramis et al. | 2004 | European | 140 | 28.0 | 4.5 | 112 | 28.4 | 4.3^a^ |  |  |  |
| Fukuyama et al. | 2006 | Asian | 30 | 26.0 | 4.2 | 63 | 25.5 | 3.9^a^ |  |  |  |
| Nakano et al. | 2006 | Asian | 61 | 21.4 | 2.23 | 121 | 22.4 | 2.8 | 69 | 21.5 | 2.29 |
| Sramkova et al. | 2007 | European | 61 | 23.0 | 3.4 | 56 | 23.7 | 4.2^a^ |  |  |  |
| Kotani et al. | 2008 | Asian | 79 | 22.9 | 3.1 | 153 | 22.2 | 2.6 | 66 | 22.2 | 2.5 |
| Binh et al. | 2011 | Asian | 26 | 20.9 | 2.4 | 76 | 20.5 | 2.7 | 38 | 20.0 | 2.5 |
| Nagai et al. | 2011 | Asian | 22 | 21.1 | 0.5 | 37 | 20.5 | 0.3 | 23 | 20.3 | 0.5 |
| Dhall et al. | 2012 | Asian | 30 | 28.5 | 7.0 | 53 | 28.5 | 6.0 | 13 | 30.8 | 8.3 |
| Yoneshiro et al. | 2012 | Asian | 54 | 21.5 | 0.4 | 94 | 21.6 | 0.3 | 51 | 22.6 | 0.5 |
| Brondani et al. | 2013 | European | 463 | 28.9 | 5.4 | 348 | 28.6 | 5.3 | 113 | 29.3 | 5.7 |
| ***UCP2* -866G/A** |  |  |  | G/G |  |  | G/A |  |  | A/A |  |
| Reference | Year | Ethinicity | N | Mean | SD | N | Mean | SD | N | Mean | SD |
| Esterbauer et al. | 2001 | European | 308 | 26.8 | 1.2 | 373 | 26.4 | 1.2 | 110 | 26.0 | 1.2 |
| Dalgaard et al. | 2003 | European | 833 | 27.1 | 5.8 | 1001 | 26.8 | 5.7 | 376 | 27.2 | 5.9 |
| Mancini et al. | 2003 | European | 279 | 29.8 | 9.6 | 247 | 30.5 | 10.9 | 46 | 28 | 9.8 |
| Sesti et al. | 2003 | European | 133 | 29.5 | 7.6 | 144 | 30.3 | 7.1 | 25 | 29.9 | 7.2 |
| D'Adamo et al. | 2004 | European | 469 | 29.9 | 6.0 | 463 | 30.0 | 6.5 | 114 | 30.0 | 6.2 |
| Ji et al. | 2004 | Asian | 72 | 23.2 | 4.1 | 148 | 22.7 | 3.1 | 72 | 22.6 | 3.1 |
| Le Feur et al | 2004 | European | 114 | 29.6 | 0.5 | 145 | 29.6 | 0.4 | 37 | 29.3 | 0.8 |
| Reis et al. | 2004 | European | 262 | 28.4 | 0.3 | 335 | 28.5 | 0.3 | 84 | 27.6 | 0.6 |
| Sasahara et al. | 2004 | European | 116 | 27.6 | 4.5 | 297 | 27.3 | 4.4^a^ |  |  |  |
| Bulotta et al. | 2005 | European | 516 | 29.7 | 5.6 | 461 | 29.5 | 5.7 | 96 | 29.1 | 5.6 |
| Shen et al. | 2006 | Asian | 686 | 23.8 | 0.6 | 876 | 24 | 0.7 | 296 | 24.2 | 0.8 |
| Akrami et al. | 2007 | Asian | 27 | 25.2 | 2.8 | 41 | 24.8 | 2.9 | 7.0 | 23.4 | 3.0 |
| Ochoa et al. | 2007 | European | 79 | 28.0 | 5.0 | 80 | 28.2 | 4.9 | 34 | 27.3 | 3.3 |
| Hamada et al. | 2008 | Asian | 80 | 24.0 | 3.1 | 131 | 24.0 | 3.2 | 68 | 24.2 | 2.9 |
| Heidari et al. | 2010 | Asian | 38 | 26.4 | 5.6 | 89 | 29.5 | 5.5 | 23 | 31.9 | 5.4 |
| Lapice et al. | 2010 | European | 185 | 30.9 | 5.8 | 198 | 30.5 | 5.9^b^ |  |  |  |
| Binh et al. | 2011 | Asian | 46 | 20.0 | 2.4 | 69 | 20.6 | 2.7 | 25 | 20.6 | 2.6 |
| Martinez-Hervas et al. | 2012 | European | 1788 | 27.2 | 0.1 | 326 | 27.9 | 0.3^b^ |  |  |  |
| Oguzkan-Balci et al. | 2013 | Asian | 17 | 30.8 | 5.9 | 83 | 27.2 | 4.8^a^ |  |  |  |
| ***UCP2* Ala55val** |  |  |  | Ala/Ala |  |  | Ala/Val |  |  | Val/Val |  |
| Reference | Year | Ethinicity | N | Mean | SD | N | Mean | SD | N | Mean | SD |
| Urhammer et al. | 1997 | European | 330 | 25.7 | 7.1 | 480 | 26.4 | 6.9 | 211 | 27.1 | 6.3 |
| Rosmond et al. | 2002 | European | 78 | 26.5 | 4.2 | 144 | 26.1 | 3.8 | 44 | 25.7 | 3.8 |
| Sale et al. | 2007 | European | 115 | 29.2 | 0.8 | 210 | 28.7 | 0.5 | 116 | 30.3 | 0.7 |
| Chen et al. | 2007 | Asian | 116 | 44.9 | 6.3 | 188 | 43.7 | 5.1^a^ |  |  |  |
| Lee et al. | 2008 | Asian | 266 | 24.6 | 2.8 | 469 | 24.8 | 3.04 | 237 | 24.6 | 2.9 |
| Bielinski et al. | 2008 | Mixed | 4051 | 27.2 | 5.1 | 5965 | 27.2 | 5.0 | 2040 | 27.2 | 5.0 |
| Martinez-Hervas et al. | 2012 | European | 1879 | 27.2 | 0.1 |  |  |  | 367 | 27.8 | 0.3^b^ |
| ***UCP2* Ins/Del** |  |  |  | Ins/Ins |  |  | Ins/Del |  |  | Del/Del |  |
| Reference | Year | Ethinicity | N | Mean | SD | N | Mean | SD | N | Mean | SD |
| Dalgaard et al. | 1999 | European | 1960 | 27.1 | 7.3 | 1612 | 26.9 | 6.8 | 368 | 27.7 | 7.3 |
| Evans et al. | 2000 | European | 47 | 38.0 | 13.6 | 240 | 37 | 14.4 | 316 | 35.0 | 13.2 |
| Yanovski et al. | 2000 | Mixed | 6 | 23.7 | 7.8 | 33 | 24.1 | 5.9 | 50 | 20.4 | 4.8 |
| Duarte et al. | 2003 | Asian | 980 | 32.2 | 6.1 | 30 | 33.8 | 5.2^a^ |  |  |  |
| Ochoa et al. | 2007 | European | 18 | 26.0 | 3.7 | 71 | 27.3 | 4.92 | 103 | 28.7 | 4.7 |
| Lee et al. | 2008 | Asian | 38 | 25.2 | 3.2 | 304 | 25.1 | 3.09 | 630 | 24.5 | 2.8 |
| Papazoglou et al. | 2012 | European | 96 | 44.5 | 4.4 | 62 | 43.2 | 2.5^a^ |  |  |  |
| Liu et al. | 2012 | Asian | 1159 | 24.9 | 4.8 | 367 | 25.1 | 4.5^a^ |  |  |  |
| ***UCP3* -55C/T** |  |  |  | C/C |  |  | C/T |  |  | T/T |  |
| Reference | Year | Ethinicity | N | Mean | SD | N | Mean | SD | N | Mean | SD |
| Meirhaeghe et al | 2000 | European | 518 | 25.8 | 4.4 | 281 | 25.5 | 4.7 | 35 | 25.7 | 3.7 |
| Otabe et al. | 2000 | European | 379 | 37.3 | 13.0 | 224 | 37.9 | 12.6 | 29 | 442 | 15.1 |
| Dalgaard et al. | 2001 | European | 2100 | 28.1 | 6.5 | 1479 | 28 | 6.4 | 290 | 28.5 | 6.6 |
| Alonso et al. | 2005 | European | 186 | 30.8 | 9.0 | 121 | 30.1 | 7.3^a^ |  |  |  |
| Fang et al. | 2005 | Asian | 129 | 27.4 | 4.7 | 148 | 27.4 | 5.1 | 23 | 29.5 | 4.8 |
| Cha et al. | 2006 | Asian | 99 | 31.4 | 3.9 | 99 | 30.7 | 3.4 | 16 | 32.3 | 3.7 |
| De Luis et al. | 2007 | European | 178 | 35.1 | 5.4 | 47 | 35.8 | 5.8^a^ |  |  |  |
| Hamada et al. | 2008 | Asian | 139 | 24.3 | 3.0 | 115 | 24.2 | 3.0 | 28 | 22.6 | 3.2 |

N/A: not available data. Studies that presented the results only in dominant (^a^) or recessive (^b^) genetic inheritance models.
