# Supplementary material for: Meta-Analysis Reveals the Association of Common Variants in the Uncoupling Protein (UCP) 1–3 Genes with Body Mass Index Variability
Source: PLoS One. 2014 May 7;9(5):e96411. doi: 10.1371/journal.pone.0096411 (PMC4013025; doi:10.1371/journal.pone.0096411)
Supplement: Flow Diagram S1 — PRISMA Flow Diagram. (DOC) [file pone.0096411.s007.doc]

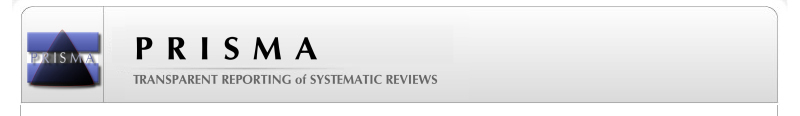
**PRISMA 2009 Flow Diagram**

**Screening**

**Included**

**Eligibility**

**Identification**

Records identified through database searching
(n = 350 )

Additional records identified through other sources
(n = 0 )

Records after duplicates removed
(n = 32 )

Records screened
(n = 318 )

Records excluded
(n = 222 )

Full-text articles assessed for eligibility
(n = 96 )

Full-text articles excluded, with reasons
(n = 40)

Studies included in qualitative synthesis
(n = 56 )

Studies included in quantitative synthesis (meta-analysis)
(n = 56 )
